# Supplementary material for: Use of tobacco and nicotine products among adolescents in Sub-Saharan Africa: protocol for a population-based multi-country household survey
Source: Front Public Health. 2025 Jul 24;13:1562352. doi: 10.3389/fpubh.2025.1562352 (PMC12330029; doi:10.3389/fpubh.2025.1562352)
Supplement: Supplementary file 1 [file Data_Sheet_1.pdf]

INDIVIDUAL QUESTIONNAIRE

The individual questionnaire will be administered to the selected eligible adolescent. The questionnaire collects information on individual characteristics and the use of tobacco and nicotine products.

MODULE 1: BACKGROUND INFORMATION

Section 1: Socio-demographic characteristics

VARNAME	QUESTIONS	CODING CATEGORIES	SKIPS	SOURCE	CORE/ OPTION-AL
**GENDER101**	Is the Respondent Male or Female?	Male = 1<br>Female = 2<br>Refused=-97		GENERAL	C
**AGE102**	How old were you at your last birthday?	Age in Completed Years __<br>don't Know Age = 98<br>Refused =- 97		ITC SUR-VEY	C
**EVESCH103**	Have you ever attended school?	Yes = 1<br>No = 2<br>Refused = -97	2 →MONEY 107	PHIA, VACS, DHS	C
**SCH104**	Are you currently enrolled in school?	Yes = 1<br>No = 2<br>Refused = -97	2 →MONEY 107	ICT-YATVS	C
**SCHTYP105**	What type of school are you currently attending?	Public/government = 1<br>Private for profit = 2<br>Private for non-profit = 3<br>International schools=4<br>Other (Specify) =96<br>Refused =- 97			
**CURGRD106**	What grade are you in now?	Grade ____<br><br>Refused = -97		GYTS, ICT-YATVS	C
**MONEIY107**	During an average week, how much money do you have that you can spend on yourself, however you want?<br><br>[ADJUST CATEGORIES FOR SPECIFIC COUNTRY]	Amount of money in the local currency<br><br>Refused = -97		GYTS	C
**ETHNIC108**	What is your ethnic/cultural / others] background?<br>[ADJUST CATEGORIES FOR SPECIFIC COUNTRY]	[ADJUST CATEGORIES FOR SPECIFIC COUNTRY]<br>OTHER = 96<br>SPECIFY:_____<br><br>REFUSED = -97		STEPs	C

VARNAME	QUESTIONS	CODING CATEGORIES	SKIPS	SOURCE	CORE/ OPTION-AL
**WORK109**	At any time during the past 12 months did you engage in any work as an employee, or self-employed individual?	Employee=1<br>Self-employed=2<br>No work=3<br>Refused=-97		PHIA	C
**RELIG110**	What is your religious affiliation [ADJUST CATEGORIES FOR SPECIFIC COUNTRY]?	01. = No religion<br>02. = Christianity;<br>03. = Islam;<br>04. = Hinduism<br>96 = Other (Specify)___<br>Refused=-97			
**MARITAL111**	What is your current marital status? Would you say you are single, married, separated, divorced, or widowed?	01. = Never married<br>02. = Married<br>03. = Living together<br>04. = Divorced<br>05. = Separated<br>06. = Widowed<br>1. -97 = Refusal to answerSingle<br>2. Married<br>3. Separated<br>4. Divorced<br>5. Widowed<br>Refused=-97		GATS	

MODULE 1: BACKGROUND INFORMATION

Section 2: Functional Difficulties
Now I am going to ask you questions about difficulties you may have doing certain activities. I will ask about difficulties seeing, hearing, walking or climbing stairs, remembering or concentrating, self-care, and communication (expressive and receptive).

**DIS101**	Do you have difficulty seeing, even if wearing glasses? (VISION)	0. No difficulty<br>1. Some difficulty<br>2. A lot of difficulty<br>3. Cannot do entirely<br>Refused=-97		WGSS	C
**DIS102**	Do you have difficulty hearing, even if using a hearing aid? (HEARING)	0. No difficulty<br>1. Some difficulty<br>2. A lot of difficulty<br>3. Cannot do entirely<br>Refused=-97		WGSS	C
**DIS103**	Do you have difficulty walking or climbing steps? (MOBILITY)	0. No difficulty<br>1. Some difficulty<br>2. A lot of difficulty<br>3. Cannot do entirely<br>Refused=-97		WGSS	C

VARNAME	QUESTIONS	CODING CATEGORIES	SKIPS	SOURCE	CORE/ OPTION-AL
**DIS104**	Do you have difficulty remembering or concentrating? (COGNITION REMEMBERING)	0. No difficulty<br>1. Some difficulty<br>2. A lot of difficulty<br>3. Cannot do entirely<br>Refused=-97		WGSS	C
**DIS105**	Do you have difficulty with self-care (such as washing all over or dressing)? (SELF-CARE)	0. No difficulty<br>1. Some difficulty<br>2. A lot of difficulty<br>3. Cannot do entirely<br>Refused=-97		WGSS	C
**DIS106**	Using your usual language, do you have difficulty communicating, for example understanding or being understood? (COMMUNICATION)	0. No difficulty<br>1. Some difficulty<br>2. A lot of difficulty<br>3. Cannot do entirely<br>Refused=-97		WGSS	C
**MODULE 2: MANUFACTURED/FACTORY- MADE CIGARETTES**					
The next section has questions that ask about smoking manufactured or factory-made cigarettes.					
**T201**	Have you ever smoked manufactured cigarettes, even once or twice??<br>(USE SHOWCARD)?	Yes = 1<br>No = 2<br>Refused =- 97	Code 2, -97→ T209	ICT-YATVS, GYTS, NYTS	C
**T202**	How old were you when you first smoked a manufactured cigarette?	Age in years__________<br>Refused =-97			C

VARNAME	QUESTIONS	CODING CATEGORIES	SKIPS	SOURCE	CORE/ OPTION-AL
**T203**	When you first smoked manufactured cigarettes, why did you smoke them? (Select one or more)	A. A friend smoked them<br>B. A family member smoked them<br>C. They costed less than other tobacco products, such as e-cigarettes<br>D. They were easier to get than other tobacco products, such as e-cigarettes<br>E. I had seen people on TV, online, or in movies smoke them<br>F. They are less harmful than other forms of tobacco, such as e-cigarettes<br>G. I could use them to do tricks<br>H. I was curious about them<br>I. Because I felt anxious, stressed, or depressed<br>J. To get a high or buzz<br>K. Packaging looks nice<br>L. Easy to hide<br>M. To get social acceptance<br>X. Other reason (specify:___________)<br>Refused =-97		NYTS/ ITC-Survey	C
**T204**	Who were you with when you first smoked manufac-tured cigarettes?	A. Alone<br>B. With one friend<br>C. With more than one friend<br>D. With a family member<br>E. With more than one family member<br>F. With a new acquaintance<br>X. Others (specify) ____----- ------------------<br>Refused=-97			
**T205**	During the past 30 days, on how many days did you smoke manufactured cigarettes?	___________days (0-30)<br>Refused=-97	Code 0, -97 → T209	ICT-YATVS, GYTS, NYTS	C

VARNAME	QUESTIONS	CODING CATEGORIES	SKIPS	SOURCE	CORE/ OPTION-AL
**T206**	During the past 30 days, on the days you smoked manufactured cigarettes, about how many manufactured cigarettes did you smoke per day?<br><br>FW: A pack usually has 20 cigarettes in it (USE SHOWCARD)?	→→→→→→→→→manufactured cigarettes per day<br>Refused =-97<br>→→→→→→→→→manufactured cigarettes per day<br>Refused =-97	→→→→→→→→→manufactured cigarettes per day<br>Refused =-97	ICT-YAT-VS,NYTS	C
**T207**	The last time you smoked manufactured cigarettes during the past 30 days, how did you get them? (SELECT ONLY ONE RESPONSE)	1. I bought them in a store or shop<br>2. I bought them from a street vendor<br>3. I bought them at a kiosk [COUNTRY-SPECIFIC]<br>4. I bought them from a vending machine [COUNTRY-SPECIFIC]<br>5. I got them from someone else<br>96. I got them some other way, specify________<br>Refused =-97		GYTS	C
**T208**	The last time you smoked manufactured cigarettes, during the past 30 days, where did you smoke them?	1. At home<br>2. At a restaurant<br>3. At a bar or club<br>4. At school<br>5. At work<br>6. At friends' houses<br>7. In public spaces (e.g. parks, shopping centers, street corners)<br>96. Other. [Please specify]<br>Refused =-97			C
**T209**	During the past 30 days, did anyone refuse to sell you manufactured cigarettes because of your age?	1. I did not try to buy manufactured cigarettes during the past 30 days.<br>2. Yes, someone refused to sell me manufactured cigarettes because of my age.<br>3. No one refused to sell me manufactured cigarettes because of my age.No, my age did not keep me from buying manufactured cigarettes.<br>Refused =-97		GYTS	C

VARNAME	QUESTIONS	CODING CATEGORIES	SKIPS	SOURCE	CORE/ OPTION-AL
**T210**	When was the last time you bought manufactured cigarettes?	1. The last 30 days<br>2. 2-3 months<br>3. 4-6 months ago<br>4. 7-12 months ago<br>5. More than 12 months ago<br>6. I have never bought manufactured cigarettes<br>Refused =-97	Code 6, -97 → TN213		C
**T211**	The last time you bought manufactured cigarettes, did you buy them as single stick(s) or pack(s)?	1. Single sticks<br>2. Pack of 10_____<br>3. Pack of 20____<br>4. Pack of 30______<br>96. Any others (specify)______<br>Refused=-97	Code -97 → TN213		C
**T212**	The last time you bought manufactured cigarettes, how much money in total did you pay for the pack containing 10 cigarettes or 20 cigarettes, or for a single stick, or any other pack size?	Amount paid_________<br><br>Refused =-97		NA-CANDA 2022-Ken-ya	C
**T213**	Do you think you will smoke manufactured cigarettes in the next 12 months?	1. Definitely yes<br>2. Probably yes<br>3. Probably not<br>4. Definitely not<br>Refused =-97		NYTS	C
**T214**	If one of your best friends were to offer you a manufac-tured cigarette, would you smoke it?	1. Definitely yes<br>2. Probably yes<br>3. Probably not<br>4. Definitely not<br>Refused =-97		NYTS	C
**T215**	If one of your close relatives were to offer you a manufac-tured cigarette, would you smoke it?	1. Definitely yes<br>2. Probably yes<br>3. Probably not<br>4. Definitely not<br>Refused =-97			C

MODULE 3: HAND-ROLLED/ROLL-YOUR-OWN (RYO) CIGARETTES

The next section has questions that ask about smoking hand-rolled or roll-your-own cigarettes. Whenever the question refers to hand-rolled cigarettes, remember that it is also referring to roll-your-own cigarettes

**T301**	Have you ever smoked hand-rolled cigarettes, even once or twice? (USE SHOWCARD)?	Yes = 1<br>No = 2<br>Refused =-97	Code 2, -97 → T309	ICT-YATVS, GYTS, NYTS	C

VARNAME	QUESTIONS	CODING CATEGORIES	SKIPS	SOURCE	CORE/ OPTION-AL
**T302**	How old were you when you first smoked a hand-rolled cigarette?	Age in years___________<br>Refused =-97			C
**T303**	When you first smoked hand-rolled cigarettes, why did you smoke them? (Select one or more)	A. A friend smoked them<br>B. A family member smoked them<br>C. They costed less than other tobacco products such as manufactured cigarettes<br>D. They were easier to get than other tobacco products such as manufactured cigarettes<br>E. I had seen people on TV, online, or in movies smoke them<br>F. They are less harmful than other tobacco products such as manufactured cigarettes<br>G. I could use them to do tricks<br>H. I was curious about them<br>I. Because I felt anxious, stressed, or depressed<br>J. To get a high or buzz<br>K. Packaging looked nice<br>L. Easier to hide<br>M. To get social acceptance<br>X. Other reason (specify:___________)<br>Refused=-97		NYTS/ ITC-Survey	C
**T304**	Who were you with when you first smoked hand rolled cigarettes?<br><br>(Multiple responses allowed)	A. Alone<br>B. With one friend<br>C. With more than one friend<br>D. With a family member<br>E. With more than one family member<br>F. With a new acquaintance<br>X. Others (specify) -----------------------<br>Refused=-97			C
**T305**	During the past 30 days, on how many days did you smoke hand-rolled cigarettes?	___________days (0-30)<br><br>Refused=-97	Code 0,-97 → T309	ICT-YATVS, GYTS, NYTS	C

VARNAME	QUESTIONS	CODING CATEGORIES	SKIPS	SOURCE	CORE/ OPTION-AL
**T306**	During the past 30 days, on the days you smoked, about how many hand-rolled cigarettes did you smoke per day?	→→→→→→→→→hand-rolled cigarettes daily<br>Refused=-97		ICT-YATVS	C
**T307**	The last time you smoked hand-rolled cigarettes during the past 30 days, how did you get them? (SELECT ONLY ONE RESPONSE)	1. I bought them in a store or shop<br>2. I bought them from a street vendor<br>3. I bought them at a kiosk [COUNTRY-SPECIFIC]<br>4. I bought them from a vending machine [COUNTRY-SPECIFIC]<br>5. I got them from someone else<br>96. I got them some other way, specify_______<br>Refused=-97		GYTS	C
**T308**	The last time you smoked hand-rolled cigarettes, during the past 30 days, where did you smoke them?	1. At home<br>2. At a restaurant<br>3. At a bar or club<br>4. At school<br>5. At work<br>6. At friends' houses<br>7. In public spaces (e.g. parks, shopping centers, street corners)<br>96.Other. [Please specify]<br>Refused=-97			C
**T309**	During the past 30 days, did anyone refuse to sell you hand-rolled cigarettes because of your age?	1. I did not try to buy hand-rolled cigarettes during the past 30 days.<br>2. Yes, someone refused to sell me hand-rolled cigarettes because of my age.<br>3. No one refused to sell me hand-rolled cigarettes because of my ageNo, my age did not keep me from buying hand rolled cigarettes.<br>Refused=-97		GYTS	C

VARNAME	QUESTIONS	CODING CATEGORIES	SKIPS	SOURCE	CORE/ OPTION-AL
**T310**	When was the last time you bought hand-rolled cigarettes?	1. The last 30 days<br>2. 2-3 months<br>3. 4-6 months ago<br>4. 7-12 months ago<br>5. More than 12 months ago<br>6. I have never bought hand-rolled cigarettes<br>Refused=-97	Code 6, -97 → TN313		C
**T311**	The last time you bought hand-rolled cigarettes, what pack size (or how much) did you buy?	___________(Pack size/ Amount/ Weight)<br><br>Refused=-97			C
**T312**	The last time you bought hand-rolled cigarettes, how much money in total did you pay?	____________(Amount)<br>Refused=-97		NA-CANDA 2022-Kenya	C
**T313**	Do you think you will smoke hand-rolled cigarettes in the next 12 months?	1. Definitely yes<br>2. Probably yes<br>3. Probably not<br>4. Definitely not<br>Refused=-97		NYTS	C
**T314**	If one of your best friends were to offer you a hand-rolled cigarette, would you smoke it?	1. Definitely yes<br>2. Probably yes<br>3. Probably not<br>4. Definitely not<br>Refused=-97		NYTS	C
**T315**	If one of your close relatives were to offer you a hand-rolled cigarette, would you smoke it?	1. Definitely yes<br>2. Probably yes<br>3. Probably not<br>4. Definitely not<br>Refused=-97			C

MODULE 4: SHISHA, WATERPIPE, OR HOOKAH

The next section has questions that ask about shisha smoking. Shisha is also known as hookah or waterpipe. Whenever the question refers to shisha, remember it is also referring to hookah or waterpipe.

VARNAME	QUESTIONS	CODING CATEGORIES	SKIPS	SOURCE	CORE/ OPTION-AL
**T401**	Have you ever smoked shisha, even one or two puffs?? (USE SHOWCARD)?	Yes = 1<br>No = 2<br>Refused = -97	Code 2, -97 → T411	GYTS, NYTS	C
**T402**	How old were you when you first smoked shisha?	Age in years___________<br>Refused=-97		GYTS, NYTS	C

VARNAME	QUESTIONS	CODING CATEGORIES	SKIPS	SOURCE	CORE/ OPTION-AL
**T403**	When you first smoked a shisha, why did you smoke it? (Select one or more)	A. A friend smoked it<br>B. A family member smoked it<br>C. To try to quit using cigarettes<br>D. It costed less than cigarettes<br>E. It was easier to get than cigarettes<br>F. I had seen people on TV, online, or in movies smoke it<br>G. It is less harmful than cigarettes<br>H. Smoking shisha may be less harmful to people around me than smoking cigarettes<br>I. It was available in flavors, such as menthol, mint, candy, fruit, or chocolate<br>J. I could smoke it unnoticed at home or at school<br>K. I could use it to do tricks<br>L. I was curious about it<br>M. Because I felt anxious, stressed, or depressed<br>N. To get a high or buzz<br>O. Packaging looked nice<br>P. To get social acceptance<br>Q. I could smoke it in places where I can't smoke cigarettes<br>R. To cut down the number of cigarettes I smoke<br>96. Other reason		NYTS/ ITC-Survey	C
**T404**	Who were you with when you first smoked shisha?<br><br>(Multiple responses allowed)	A. Alone<br>B. With one friend<br>C. With more than one friend<br>D. With a family member<br>E. With more than one family member<br>F. With a new acquaintance<br>X.Others (specify) -----------------------<br>Refused=-97			C
**T405**	During the past 30 days, on how many days did you smoke shisha?	__________days (0-30)<br>Refused=-97	Code 0, -97 →T411	GYTS, NYTS	C

VARNAME	QUESTIONS	CODING CATEGORIES	SKIPS	SOURCE	CORE/ OPTION-AL
**T406**	During the past 30 days, on the days you smoked shisha, about how many shisha sessions did you participate in on a typical shisha smoking day?<br>(USE SHOWCARD)?	→→→→→→→→→shisha sessions on a typical shisha smoking day<br>Refused=-97		GATS	C
**T407**	The last time you smoked shisha during the past 30 days, how did you get it?	1. I bought it in a store or shop<br>2. I bought it from a street vendor<br>3. I bought it at a kiosk [COUNTRY-SPECIFIC]<br>4. I bought it from a vending machine [COUNTRY-SPECIFIC]<br>5. I got it from someone else<br>6. I bought it from a restaurant/bar/club<br>96. I got it some other way, specify<br>Refused=-97			C
**T408**	Was any of the shisha that you smoked in the past 30 days flavored to taste like menthol, mint, clove or spice, alcoholic drinks, candy, fruit, chocolate, or any other flavor?	Yes = 1<br>No = 2<br>Refused =-97		NYTS	C
**T409**	Was any of the shisha that you smoked in the past 30 days mixed with other drugs such as bhang/marijuana?	Yes = 1<br>No = 2<br>Refused = -97			C
**T410**	The last time you smoked shisha during the past 30 days, where did you smoke it?	1. At home<br>2. At a restaurant<br>3. At a bar or club<br>4. At school<br>5. At work<br>6. At friends' houses<br>7. In public spaces (e.g. parks, shopping centers, street corners)<br>96.Other. [Please specify]<br>Refused=-97		Nigeria survey, GYTS	C

VARNAME	QUESTIONS	CODING CATEGORIES	SKIPS	SOURCE	CORE/ OPTION-AL
**T411**	During the past 30 days, did anyone refuse to sell you shisha because of your age?	1. I did not try to buy shisha during the past 30 days.<br>2. Yes, someone refused to sell me shisha because of my age.<br>3. No one refused to sell me shisha because of my age.No, my age did not keep me from buying shisha.<br>Refused=-97		GYTS	C
**T412**	When was the last time you bought shisha?	1. The last 30 days<br>2. 2-3 months<br>3. 4-6 months ago<br>4. 7-12 months ago<br>5. More than 12 months ago<br>6. I have never bought shisha<br>Refused=-97	Code 6, -97 →T414		C
**T413**	The last time you bought shisha, how much in total did you buy?<br>A. Complete hookah setup (a Hookah Pipe, a Hose, A Bowl, Shisha Tobacco, Charcoals, Tongs, Tin Foil or a Heat Management Device, Something to Light Your Charcoal)	____________/Complete hookah setup<br>Refused=-97			C
	B. OR Shisha session	____________/Shisha session<br>Refused=-97			
	C. OR Shisha Pot	____________/Shisha Pot<br>Refused=-97			
	D. OR Per gram	____________/gram<br>Refused=-97			
	E. OR Others (specify)__	____________/Others (specify)<br>Refused=-97			
**T414**	Do you think you will smoke shisha in the next 12 months?	1. Definitely yes<br>2. Probably yes<br>3. Probably not<br>4. Definitely not<br>Refused=-97		NYTS	C

VARNAME	QUESTIONS	CODING CATEGORIES	SKIPS	SOURCE	CORE/ OPTION-AL
**T415**	If one of your best friends were to offer you shisha, would you smoke it?	1. Definitely yes<br>2. Probably yes<br>3. Probably not<br>4. Definitely not<br>Refused=-97		NYTS	C
**T416**	If one of your close relatives were to offer you shisha would you smoke it?	1. Definitely yes<br>2. Probably yes<br>3. Probably not<br>4. Definitely not<br>Refused=-97			C

MODULE 5: HEATED TOBACCO PRODUCTS

The next section has questions that ask about heated tobacco products. These devices heat actual tobacco (in sticks or capsules) to create an aerosol that is inhaled. These are NOT THE SAME as e-cigarettes, which use liquids. Heated tobacco products include brands like IQOS ([CA/UK: HEETS; US: Marlboro HeatSticks])

VARNAME	QUESTIONS	CODING CATEGORIES	SKIPS	SOURCE	CORE/ OPTION-AL
**HTP501**	Do you own a heated tobacco device (SHOWCARD)?	Yes = 1<br>No = 2<br>Refused = -97	Code 1, -97 → HTP504	ICT-YATVS	C
**HTP502**	Before this survey, had you seen or heard of heated tobacco products? (USE SHOWCARD)	Yes = 1<br>No = 2<br>Refused =- 97	Code 2, -97 → HTP504	ICT-YATVS	C
**HTP503**	Where did you first see or hear about heated tobacco products?	1. Traditional media: Television, radio, newspapers<br>2. The internet (online malls, news and non-news web pages)<br>3. Social media (Facebook, twitter, Instagram, WhatsApp groups)<br>4. Friends<br>5. Social gatherings (parties, lounges)<br>96.Others specify______<br>Refused=-97		Nigeria survey	C
**HTP504**	Have you ever smoked heated tobacco products, even one or two puffs? (USE SHOWCARD)	Yes = 1<br>No = 2<br>Refused = -97	Code 2, -97 → HTP514	ICT-YATVS	C
**HTP505**	How old were you when you first smoked heated tobacco products?	Age in years___________<br>Refused=-97		ICT-YATVS	C

VARNAME	QUESTIONS	CODING CATEGORIES	SKIPS	SOURCE	CORE/ OPTION-AL
**HTP506**	When you first smoked heated tobacco products, why did you smoke them? (Select one or more)	A. A friend smoked them<br>B. A family member smoked them<br>C. To try to quit smoking cigarettes<br>D. They costed less than cigarettes<br>E. They were easier to get than cigarettes<br>F. I had seen people on TV, online, or in movies smoking them<br>G. They are less harmful than cigarettes<br>H. Smoking heated tobacco may be less harmful to people around me than smoking cigarettes<br>I. They were available in flavors, such as menthol, mint, candy, fruit, or chocolate<br>J. I could smoke them unnoticed at home or at school<br>K. I could use them to do tricks<br>L. I was curious about them<br>M. Because I felt anxious, stressed, or depressed<br>N. To get a high or buzz<br>O. Packaging looks nice<br>P. To get social acceptance<br>Q. I could smoke them in places where I can't smoke cigarettes<br>R. To cut down the number of cigarettes I smoke<br>X. Other		NYTS/ ITC-Survey	C
**HTP507**	Who were you with when you first smoked a heated tobacco product?<br><br>(Multiple responses allowed)	A. Alone<br>B. With one friend<br>C. With more than one friend<br>D. With a family member<br>E. With more than one family member<br>F. With a new acquaintance<br>X.Others (specify) -----------------------<br>Refused=-97			C

VARNAME	QUESTIONS	CODING CATEGORIES	SKIPS	SOURCE	CORE/ OPTION-AL
**HTP508**	What specific variety of heated tobacco product do you smoke most often? [ADJUST CATEGORIES FOR SPECIFIC COUNTRY]	1. Marlboro Original (silver)<br>2. Marlboro Fresh Menthol<br>3. Marlboro Smooth Menthol<br>4. Amber<br>5. Yellow<br>6. Sienna<br>7. Mauve<br>8. Russet<br>9. Teak<br>10. Green (menthol)<br>11. Turquoise (menthol)<br>12. Blue (menthol)<br>13. Other (Please specify:)______<br>14. I don't use a specific variety more often than others<br><br>I don't know the type=-98<br>Refused=-97<br>[ADJUST CATEGORIES FOR SPECIFIC COUNTRY]		ICT-YATVS	C
**HTP509**	During the past 30 days, on how many days did you smoke heated tobacco products?	___________days (0-30)<br>Refused=-97	Code 0, -97 → HTP514	ICT-YATVS	C
**HTP510**	During the past 30 days, on the days you smoked a heated tobacco product, about how many times did you smoke it each day?	1. 1 time per day<br>2. 2 to 5 times per day<br>3. 6 to 10 times per day<br>4. 11 to 20 times per day<br>5. More than 20 times per day<br>6. Don't know<br>Refused=-97		ITC	C
**HTP511**	What brand of heated tobacco product did you smoke most often in the past 30 days?	1. IQOS<br>2. glo<br>3. Ploom<br>4. iFuse<br>5. lil<br>6. Other (Please specify:)______<br>7. I don't have a usual brand<br>I don't know the brand=-98<br>Refused=-97		ICT-YATVS	C

VARNAME	QUESTIONS	CODING CATEGORIES	SKIPS	SOURCE	CORE/ OPTION-AL
**HTP512**	What flavors were the heated tobacco products that you have smoked in the past 30 days? (Select one or more)	A. Menthol<br>B. Mint<br>C. Clove or spice<br>D. Fruit<br>E. Chocolate<br>F. Alcoholic drinks (such as wine, margarita, or other cocktails)<br>G. Candy, desserts, or other sweets<br>96. Some other flavor not listed here (Specify:______)<br>Refused=-97		NYTS	C
**HTP513**	The last time you smoked heated tobacco products during the past 30 days, where did you smoke them?	1. At home<br>2. At a restaurant<br>3. At a bar or club<br>4. School<br>5. At work<br>6. At friends' houses<br>7. In public spaces (e.g. parks, shopping centers, street corners)<br>96.Other. [Please specify]<br>Refused=-97		GYTS	C
**HTP514**	During the past 30 days, did anyone refuse to sell you heated tobacco products because of your age?	1. I did not try to buy heated tobacco products during the past 30 days.<br>2. Yes, someone refused to sell me heated tobacco products because of my age.<br>3. No one refused to sell me heated tobacco products because of my ageNo, my age did not keep me from buying heated tobacco products.<br>Refused =-97		GYTS	C

VARNAME	QUESTIONS	CODING CATEGORIES	SKIPS	SOURCE	CORE/ OPTION-AL
**HTP515**	When you smoked heated tobacco products during the past 30 days, how did you get your heated tobacco products? (Select one or more)	A. I bought them myself<br>B. I had someone else buy them for me<br>C. I asked someone to give me some<br>D. Someone offered them to me<br>E. I got them from a friend<br>F. I got them from a family member<br>G. I got them from a store or another person<br>X. I got them in some other way (specify: ___________)<br>Refused=-97		NYTS	C
**HTP516**	If you bought these products, how much did you spend for a full package?	______________/full package<br>Refused=-97			C
**HTP517**	During the past 30 days, where did you buy heated tobacco products and devices? (Select one or more)	A. I did not buy heated tobacco products during the past 30 days [EXCLUSIVE RESPONSE]<br>B. I bought them from another person (a friend, family member, or someone else)<br>C. A gas station or convenience store<br>D. A grocery store<br>E. A drugstore<br>F. A mall or shopping center kiosk/stand<br>G. A vending machine<br>H. On the Internet (such as a product website or store website like eBay or Facebook Marketplace)<br>I. Through the mail<br>J. Through a delivery service (such as DoorDash or Postmates)<br>K. A vape shop or tobacco shop<br>X. Some other place not listed here (specify):___________<br>Refused=-97		NYTS	C

VARNAME	QUESTIONS	CODING CATEGORIES	SKIPS	SOURCE	CORE/ OPTION-AL
**HTP518**	Do you think you will smoke heated tobacco products in the next 12 months?	1. Definitely yes<br>2. Probably yes<br>3. Probably not<br>4. Definitely not<br>Refused=-97		NYTS	C
**HTP519**	If one of your best friends were to offer you a heated tobacco product, would you smoke it?	1. Definitely yes<br>2. Probably yes<br>3. Probably not<br>4. Definitely not<br>Refused=-97		ICT-YATVS	C
**HTP520**	If one of your close relatives were to offer you a heated tobacco product, would you smoke it?	1. Definitely yes<br>2. Probably yes<br>3. Probably not<br>4. Definitely not<br>Refused=-97		ICT-YATVS	C

MODULE 6: OTHER SMOKED TOBACCO PRODUCTS

The next section has questions that ask about smoking other tobacco products such as cigars, cheroots, cigarillos. These exclude manufactured, hand-rolled cigarettes, shisha, or heated tobacco products which have already been explored.

**T601**	Have you ever smoked other tobacco products such as cigars/cheroots/cigarillos, even once or twice?<br><br>(USE SHOWCARD)	Yes = 1<br>No = 2<br>Refused =- 97	Code 2 →T609	ICT-YATVS, GYTS, NYTS	C
**T602**	How old were you when you first smoked other tobacco products such as cigars/che-roots/cigarillos?	Age in years___________<br>Refused=-97			C

VARNAME	QUESTIONS	CODING CATEGORIES	SKIPS	SOURCE	CORE/ OPTION-AL
**T603**	When you first smoked other tobacco products such as cigars/cheroots/cigarillos, why did you smoke them ? (Select one or more)	A. A friend smoked them<br>B. A family member smoked them<br>C. To try to quit smoking cigarettes<br>D. They costed less than cigarettes<br>E. They were easier to get than cigarettes<br>F. I had seen people on TV, online, or in movies smoking them<br>G. They are less harmful than cigarettes<br>H. Smoking other tobacco products such as cigars, cheroots or cigarillos may be less harmful to people around me than smoking cigarettes<br>I. They were available in flavors, such as menthol, mint, candy, fruit, or chocolate<br>J. I could smoke them unnoticed at home or at school<br>K. I could use them to do tricks<br>L. I was curious about them<br>M. Because I felt anxious, stressed, or depressed<br>N. To get a high or buzz<br>O. Packaging looks nice<br>P. To get social acceptance<br>Q. I could smoke them in places where I can't smoke cigarettes<br>R. To cut down the number of cigarettes I smoke<br>X. Other		NYTS/ ITC-Survey	C
**T604**	Who were you with when you first smoked other tobacco products (such as cigars/cheroots/cigarillos)?<br><br>(Multiple responses allowed)	A. Alone<br>B. With one friend<br>C. With more than one friend<br>D. With a family member<br>E. With more than one family member<br>F. With a new acquaintance<br>X.Others (specify) -----------------------<br>Refused=-97			C

VARNAME	QUESTIONS	CODING CATEGORIES	SKIPS	SOURCE	CORE/ OPTION-AL
**T605**	During the past 30 days, on how many days did you smoke other tobacco products such as cigars/cheroots/ cigarillos?	___________days (0-30)<br>Refused=-97	Code 0 →T609	ICT-YATVS, GYTS, NYTS	C
**T606**	During the past 30 days, on the days you smoked other tobacco products such as cigars/cheroots/cigarillos, about how many did you smoke per day?<br><br>(USE SHOWCARD)?	→→→→→→→→→cigars/day<br><br>→→→→→→→→→cheroots/day<br><br>→→→→→→→→→cigarillos/day<br><br>→→→→→→→→→other tobacco products (specify)/day<br>Refused=-97		ICT-YATVS	C
**T607**	The last time you smoked other tobacco products such as cigars/cheroots/cigarillos during the past 30 days, how did you get them?<br><br>(SELECT ONLY ONE RE-SPONSE)	1. I bought them in a store or shop<br>2. I bought them from a street vendor<br>3. I bought them at a kiosk [COUNTRY-SPECIFIC]<br>4. I bought them from a vending machine [COUNTRY-SPECIFIC]<br>5. I got them from someone else<br>96. Other way specify_______<br>Refused=-97		GYTS	C
**T608**	The last time you smoked other tobacco products, during the past 30 days, where did you smoke them?	1. At home<br>2. At a restaurant<br>3. At a bar or club<br>4. At school<br>5. At work<br>6. At friends' houses<br>7. In public spaces (e.g. parks, shopping centers, street corners)<br>96. Other. [Please specify]<br>Refused=-97			C

VARNAME	QUESTIONS	CODING CATEGORIES	SKIPS	SOURCE	CORE/ OPTION-AL
**T609**	During the past 30 days, did anyone refuse to sell you other smoked tobacco products such as cigars/ cheroots/cigarillos because of your age?	1. I did not try to buy other smoked tobacco products during the past 30 days.<br>2. Yes, someone refused to sell me other smoked tobacco products because of my age.<br>3. No one refused to sell me other smoked tobacco products because of my ageNo, my age did not keep me from buying other smoked tobacco products.<br>Refused=-97		GYTS	C
**T610**	When was the last time you bought other smoked tobacco products such as cigars/ cheroots/cigarillos?	1. The last 30 days<br>2. 2-3 months<br>3. 4-6 months ago<br>4. 7-12 months ago<br>5. More than 12 months ago<br>6. I have never bought other tobacco products such as cigars<br>Refused=-97	Code 6 →TN613		C
**T611**	The last time you bought other smoked tobacco products such as cigars/cheroots/ cigarillos, did you buy them as a rod or pack(s)?	1. Rods<br>2. Packs<br>Refused =-97			C
**T612**	The last time you bought other smoked tobacco products such as cigars/cheroots/ cigarillos, how much did you spend for a rod/ pack?	____________/cigar rod pack<br><br>____________/cheroots rod/pack<br><br>____________/cigarillos rod/pack<br><br>____________/other smoked tobacco product (specify) rod/pack<br>Refused=-97			C
**T613**	Do you think you will smoke other tobacco products such as cigars/cheroots/cigarillos in the next 12 months?	1. Definitely yes<br>2. Probably yes<br>3. Probably not<br>4. Definitely not<br>Refused=-97		NYTS	C

VARNAME	QUESTIONS	CODING CATEGORIES	SKIPS	SOURCE	CORE/ OPTION-AL
**T614**	If one of your best friends were to offer you other smoked tobacco products such as cigars/cheroots/ cigarillos, would you smoke them?	1. Definitely yes<br>2. Probably yes<br>3. Probably not<br>4. Definitely not<br>Refused=-97		NYTS	C
**T615**	If one of your close relatives were to offer you other smoked tobacco products such as cigars/cheroots/ cigarillos would you smoke them?	1. Definitely yes<br>2. Probably yes<br>3. Probably not<br>4. Definitely not<br>Refused=-97			C

MODULE 7: SMOKELESS TOBACCO

The next section has questions that ask about smokeless tobacco use. This includes (FILL AS APPROPRIATE): i) chewing tobacco such as tobacco leaf, tobacco leaf and lime, ii) applying tobacco such as, tobacco toothpaste-dentobac etc.; tobacco tooth powder-lal, etc.; snuff).

VARNAME	QUESTIONS	CODING CATEGORIES	SKIPS	SOURCE	CORE/ OPTION-AL
**ST701**	Have you ever used smoke-less tobacco products such as chewing tobacco, snuff, or dip, even just a small amount once or twice?<br>(USE SHOWCARD)?	Yes = 1<br>No = 2<br>Refused = 97	Code 2 →ST710	GYTS, NYTS	C
**ST702**	How old were you when you first used smokeless tobacco such as chewing tobacco, snuff, or dip?	Age in years__________<br>Refused=-97		GYTS, NYTS	C
**ST703**	When you first used smoke-less tobacco products such as chewing tobacco, snuff, or dip, why did you use them ?<br><br>(Select one or more)	A. A friend uses them<br>B. A family member uses them<br>C. They costed less than cigarettes<br>D. They were easier to get than cigarettes<br>E. I had seen people on TV, online, or in movies use them<br>F. They are less harmful than other forms of tobacco, cigarettes<br>G. I could use them to do tricks<br>H. I was curious about them<br>I. Because I felt anxious, stressed, or depressed<br>J. To get a high or buzz<br>K. Packaging looked nice<br>L. Easy to hide<br>M. To get social acceptance<br>X. Other reason		NYTS/ ITC-Survey	C

VARNAME	QUESTIONS	CODING CATEGORIES	SKIPS	SOURCE	CORE/ OPTION-AL
**ST704**	Who were you with when you first used a smokeless tobacco product?<br><br>(Multiple responses allowed)	A. Alone<br>B. With one friend<br>C. With more than one friend<br>D. With a family member<br>E. With more than one family member<br>F. With a new acquaintance<br>X.Others (specify) -----------------------<br>Refused=-97			
**ST705**	During the past 30 days, on how many days did you use smokeless tobacco such as chewing tobacco, snuff, or dip?	___________days (0-30)<br>Refused=-97	Code 0, -97 ->ST710	GYTS, NYTS	C
**ST706**	During the past 30 days, on the days you used smokeless tobacco such as chewing tobacco, snuff, or dip, about how many times did you use it per day?	→→→→→→→→→times/day<br>Refused=-97			C
**ST707**	The last time you used smokeless tobacco such as chewing tobacco, snuff, or dip during the past 30 days, how did you get them?<br><br>(SELECT ONLY ONE RE-SPONSE)	1. I did not use smokeless tobacco such as chewing tobacco, snuff, or dip products during the past 30 days<br>2. I bought them in a store or shop<br>3. I bought them from a street vendor<br>4. I bought them at a kiosk [COUNTRY-SPECIFIC]<br>5. I bought them from a vending machine [COUNTRY-SPECIFIC]<br>6. I got them from someone else<br>96. I got them some other way. Specify_____<br>Refused=-97		GYTS	C
**ST708**	Was any of the smokeless tobacco products such as chewing tobacco, snuff, or dip that you used in the past 30 days flavored to taste like menthol, mint, clove or spice, alcoholic drinks, candy, fruit, chocolate, or any other flavor?	Yes = 1<br>No = 2<br>Refused =- 97		NYTS	C

VARNAME	QUESTIONS	CODING CATEGORIES	SKIPS	SOURCE	CORE/ OPTION-AL
**ST709**	The last time you used smokeless tobacco products, during the past 30 days, where did you use them?	1. At home<br>2. At a restaurant<br>3. At a bar or club<br>4. At school<br>5. At work<br>6. At friends' houses<br>7. In public spaces (e.g. parks, shopping centers, street corners)<br>96. Other. [Please specify]<br>Refused=-97			C
**ST710**	During the past 30 days, did anyone refuse to sell you smokeless tobacco products such as chewing tobacco, snuff, or dip because of your age?	1. I did not try to buy smokeless tobacco products during the past 30 days.<br>2. Yes, someone refused to sell me smokeless tobacco products because of my age.<br>3. No one refused to sell me smokeless tobacco products because of my age. No, my age did not keep me from buying smokeless tobacco products.<br>Refused=-97		GYTS	C
**ST711**	When was the last time you bought smokeless tobacco products such as chewing tobacco, snuff, or dip?	1. The last 30 days<br>2. 2-3 months<br>3. 4-6 months ago<br>4. 7-12 months ago<br>5. More than 12 months ago<br>6. I have never bought smokeless tobacco products such as chewing tobacco, snuff, or dip<br>Refused=-97	Code 6 ->STN714		C
**ST712**	The last time you bought smokeless tobacco products, what pack size (or how much) did you buy?	____________(Pack size/ QuantityAmount)<br><br>Refused=-97			C
**ST713**	The last time you bought smokeless tobacco products, how much money in total did you pay?	____________(Amount)<br><br>Refused=-97		NA-CANDA 2022-Kenya	C

VARNAME	QUESTIONS	CODING CATEGORIES	SKIPS	SOURCE	CORE/ OPTION-AL
**ST714**	Do you think you will use smokeless tobacco in the next 12 months?	1. Definitely yes<br>2. Probably yes<br>3. Probably not<br>4. Definitely not<br>Refused=-97		NYTS	C
**ST715**	If one of your best friends were to offer you smokeless tobacco, would you use it?	1. Definitely yes<br>2. Probably yes<br>3. Probably not<br>4. Definitely not<br>Refused=-97		NYTS	C
**ST716**	If one of your close relatives were to offer you smokeless tobacco, would you use it?	1. Definitely yes<br>2. Probably yes<br>3. Probably not<br>4. Definitely not<br>Refused=-97			C
**MODULE 8: ELECTRONIC CIGARETTES MODULE**<br>The next section has questions that ask about electronic cigarettes, or e-cigarettes. Electronic cigarettes are electronic devices that usually contain a nicotine-based liquid that is vaporized and inhaled. You may also know them as vapes, vape-pens, hookah-pens, electronic hookahs (e-hookahs), electronic cigars (e-cigars), electronic pipes (e-pipes), or e-vaporizers. Some look like cigarettes and others look like pens or small pipes. These are battery-powered devices that produce vapor instead of smoke.					
**ET801**	Before today, had you ever seen or heard of electronic cigarettes or e-cigarettes such as JUUL, SMOK, Suorin, Vuse, blu, Puff Bar, or STIG? (USE SHOWCARD)	Yes = 1<br>No = 2<br>Refused =-97	Code 2, -97 ->ET803	GYTS	C
**ET802**	Where did you first see or hear about electronic cigarettes such as JUUL, SMOK, Suorin, Vuse, blu, Puff Bar, or STIG?	1. Traditional media: Television, radio, newspapers?<br>2. The internet (online malls, news and non-news web pages)?<br>3. Social media (Facebook, twitter, Instagram, WhatsApp groups)?<br>4. Friends?<br>5. Social gatherings (parties, lounges)<br>96.Other specify_____<br>Refused=-97		Nigeria survey	C

VARNAME	QUESTIONS	CODING CATEGORIES	SKIPS	SOURCE	CORE/ OPTION-AL
**ET803**	Have you ever smoked an electronic cigarette, or e-cig-arette such as JUUL, SMOK, Suorin, Vuse, blu, Puff Bar, or STIG, even once or twice?<br><br>(USE SHOWCARD)<br><br>FW note: Ask for all the responses in ET801	Yes = 1<br>No = 2<br>Refused = -97	Code 2, -97->ET8123	ICT-YATVS, GYTS, NYTS	C
**ET804**	How old were you when you first smoked an electronic cigarette?	Age in years__________<br>Refused=-97			C

VARNAME	QUESTIONS	CODING CATEGORIES	SKIPS	SOURCE	CORE/ OPTION-AL
**ET805**	When you first smoked electronic cigarettes, why did you smoke them?<br><br>(Select one or more)	A. A friend smoked them<br>B. A family member smoked them<br>C. To try to quit smoking cigarettes<br>D. They costed less than cigarettes<br>E. They were easier to get than cigarettes<br>F. I had seen people on TV, online, or in movies smoking them<br>G. They are less harmful than cigarettes<br>H. Smoking electronic cigarettes may be less harmful to people around me than smoking cigarettes<br>I. They were available in flavors, such as menthol, mint, candy, fruit, or chocolate<br>J. I could smoke them unnoticed at home or at school<br>K. I could use them to do tricks<br>L. I was curious about them<br>M. Because I felt anxious, stressed, or depressed<br>N. To get a high or buzz<br>O. Packaging looks nice<br>P. To get social acceptance<br>Q. I could smoke them in places where I can't smoke cigarettes<br>R. To cut down the number of cigarettes I smoke<br>X. Other reasons (specify:________)<br>Refused=-97		NYTS/ ITC-Survey	C

VARNAME	QUESTIONS	CODING CATEGORIES	SKIPS	SOURCE	CORE/ OPTION-AL
**ET806**	Who were you with when you first smoked an electronic cigarette?<br><br>(Multiple responses allowed)	A. Alone<br>B. With one friend<br>C. With more than one friend<br>D. With a family member<br>E. With more than one family member<br>F. With a new acquaintance<br>X.Others (specify)<br>-----------------------<br>Refused=-97			C
**ET807**	During the past 30 days, on how many days did you smoke an electronic cigarette?	___________days (0-30)<br>Refused=-97	Code 0,-97 → ET813	GYTS/ NYTS	C
**ET808**	During the past 30 days, on the days you smoked an electronic cigarette, about how many times did you use it each day?	1. 1 time per day<br>2. 2 to 5 times per day<br>3. 6 to 10 times per day<br>4. 11 to 20 times per day<br>5. More than 20 times per day<br>6. Don't know<br>Refused=-97		ITC	C
**ET809**	Which of the following best describes the type of electronic cigarette you have smoked in the past 30 days?<br><br>If you have used more than one type, please think about the one you use most often.	1. A disposable electronic cigarette (for example, Puff Bar or STIG)<br>2. An electronic cigarette that uses pre-filled or refillable pods or cartridges (for example, JUUL, SMOK, or Suorin)<br>3. An electronic cigarette with a tank that you refill with liquids (including mod systems that can be customized by the user)<br>4. I don't know the type		NYTS	C
**ET810**	Were any of the electronic cigarettes that you smoked in the past 30 days flavored to taste like menthol, mint, clove or spice, alcoholic drinks, candy, fruit, chocolate, or any other flavor?	Yes = 1<br>No = 2<br>Refused = -97	Code 2, -97 → ET812	NYTS	C

VARNAME	QUESTIONS	CODING CATEGORIES	SKIPS	SOURCE	CORE/ OPTION-AL
**ET811**	What flavors were the electronic cigarettes that you have smoked in the past 30 days?<br><br>(Select one or more)	A. Menthol<br>B. Mint<br>C. Clove or spice<br>D. Fruit<br>E. Chocolate<br>F. Alcoholic drinks (such as wine, margarita, or other cocktails)<br>G. Candy, desserts, or other sweets<br>X. Some other flavor not listed here (Specify:______)<br>Refused=-97		NYTS	C
**ET812**	The last time you smoked an electronic cigarette during the past 30 days, where did you smoke it?	1. At home<br>2. At a restaurant<br>3. At a bar or club<br>4. School<br>5. At work<br>6. At friends' houses<br>7. In public spaces (e.g. parks, shopping centers, street corners)<br>96. Other. [Please specify]<br>Refused=-97		Nigeria survey, GYTS	C
**ET813**	During the past 30 days, did anyone refuse to sell you an electronic cigarette device, pods, cartridges, or e-liquid refills because of your age?	1. I did not try to buy an electronic cigarette device, pods, cartridges, or e-liquid refills during the past 30 days.<br>2. Yes, someone refused to sell me an electronic cigarette device, pods, cartridges, or e-liquid refills because of my age.<br>3. No one refusedNo refused to sell me , my age did not keep me from buying an electronic cigarette device, pods, cartridges, or e-liquid refills because of my age.<br>Refused =-97		GYTS	C

VARNAME	QUESTIONS	CODING CATEGORIES	SKIPS	SOURCE	CORE/ OPTION-AL
**ET814**	During the past 30 days, how did you get electronic cigarette devices, pods, cartridges, or e-liquid refills?<br><br>(Select one or more)	A. I bought them myself<br>B. I had someone else buy them for me<br>C. I asked someone to give me some<br>D. Someone offered them to me<br>E. I got them from a friend<br>F. I got them from a family member<br>G. I got them from a store or another person<br>X. I got them in some other way (specify: ___________)<br>Refused=-97		NYTS	C
**ET815**	If you bought these products, how much did you spend for a full package?	_____________/full package (in local currency)<br>Refused=-97			C
**ET816**	During the past 30 days, where did you buy your electronic cigarette devices, pods, cartridges, or e-liquid refills?<br><br>(Select one or more)	A. I did not buy e-cigarettes during the past 30 days [EXCLUSIVE RESPONSE]<br>B. I bought them from another person (a friend, family member, or someone else)<br>C. A gas station or convenience store<br>D. A grocery store<br>E. A drugstore<br>F. A mall or shopping center kiosk/stand<br>G. A vending machine<br>H. On the Internet (such as a product website or store website like eBay or Facebook Marketplace)<br>I. Through the mail<br>J. Through a delivery service (such as DoorDash or Postmates)<br>K. A vape shop or tobacco shop<br>X. Some other place not listed here (specify):___________<br>Refused=-97		NYTS	C

VARNAME	QUESTIONS	CODING CATEGORIES	SKIPS	SOURCE	CORE/ OPTION-AL
**ET817**	Do you think you will smoke electronic cigarettes in the next 12 months?	1. Definitely yes<br>2. Probably yes<br>3. Probably not<br>4. Definitely not<br>Refused=-97		NYTS	C
**ET818**	If one of your best friends were to offer you an electronic cigarette, would you smoke it?	1. Definitely yes<br>2. Probably yes<br>3. Probably not<br>4. Definitely not<br>Refused=-97		NYTS	C
**ET819**	If one of your close relatives were to offer you an electronic cigarette, would you smoke it?	1. Definitely yes<br>2. Probably yes<br>3. Probably not<br>4. Definitely not<br>Refused=-97			C
**MODULE 9: Knowledge, Attitudes, Perceptions, Intentions**					
**KAPI901**	Are your parents/legal guardians aware that you use a tobacco product?	1.Yes<br>2.No<br>3. Don't know<br><br>Refused=-97	Not applicable for those who responded no to all of the following: T201; T301; T401; HTP504; T601; ST701; ET803		C
**KAPI902**	Do any of your family members, relatives, /tutor/teacher, or neighbor use tobacco products?<br><br>(More than one response allowed)	A. None<br>B. Father<br>C. Mother<br>D. Tutor<br>E. Teacher<br>F. Sister<br>G. Brother<br>H. Another family member<br>I. Neighbor<br>J. Don't know<br>X. Others specify__________<br>Refused=-97		GYTS	C
**KAPI903**	Do any of your closest friends use tobacco?	1. None of them<br>2. Some of them<br>3. Most of them<br>4. All of them<br>Don't know=-98<br>Refused=-97		GYTS	C
**KAPI904**	How many young people aged between 10 and 17 in your immediate circle consume tobacco products?	__________young people<br>Refused=-97<br>Don't know=-98			C

VARNAME	QUESTIONS	CODING CATEGORIES	SKIPS	SOURCE	CORE/ OPTION-AL
**KAPI905**	Do you think using tobacco makes young people look more or less attractive?	1. More attractive<br>2. Less attractive<br>3. No difference from non-smokers<br>Refused=-97<br>Don't know=-98		GYTS	C
**KAPI906**	Do you think using tobacco is harmful to your health?	1. Definitely not<br>2. Probably not<br>3. Probably yes<br>4. Definitely yes<br>I don't know=-98<br>Refused=-97		GYTS	C
**KAPI907**	Do you think it is safe to use tobacco for only a year or two as long as you quit after that?	1. Definitely not<br>2. Probably not<br>3. Probably yes<br>4. Definitely yes<br>I don't know=-98<br>Refused=-97		GYTS	C
**KAPI908**	Do you think there are safe tobacco products?	1. Yes<br>2. No<br>I don't know=-98<br>Refused=-97	Code 2, -98 or -97 →KAPI910		C
**KAPI909**	What tobacco products are safe (mention all the products)?				C
**KAPI910**	During the past 12 months, did you read in your school texts or books about the health effects of tobacco?	1. Yes<br>2. No<br>3. I do not have school texts or books<br>Refused=-97		GYTS	C
**KAPI911**	Do you think the smoke from other people's tobacco smoking is harmful to you?	1. Definitely not<br>2. Probably not<br>3. Probably yes<br>4. Definitely yes<br>I don't know= -98<br>Refused=-97		GYTS	C
**KAPI912**	Do you think smoking tobacco helps people feel more comfortable or less comfortable at celebrations, parties, or in other social gatherings?	1. More comfortable<br>2. Less comfortable<br>3. No difference whether smoking or not<br>Refused=-97		GYTS	C
**KAPI913**	On average, how much do you think a pack of 20 manufactured cigarettes costs?	____________Cost (in local currency)<br><br>I don't know=-98<br>Refused=-97		GYTS	C

VARNAME	QUESTIONS	CODING CATEGORIES	SKIPS	SOURCE	CORE/ OPTION-AL
**KAPI914**	During the past 30 days, did you see or hear any anti-tobacco media messages on television, radio, internet, billboards, posters, newspapers, magazines, movies?	1. Yes<br>2. No<br>Refused=-97		GYTS	C
**KAPI915**	During the past 30 days, did you see or hear any anti-tobacco media messages on social media platforms such as Instagram, Facebook, WhatsApp, TikTok, Twitter, LinkedIn, Pinterest, YouTube, and Snapchat?	1. Yes<br>2. No<br>Refused=-97		GYTS	C
**KAPI916**	During the past 30 days, did you see any people using tobacco on TV?	1. Yes<br>2. No<br>Refused=-97		GYTS	C
**KAPI917**	During the past 30 days, did you see any people using tobacco in social media platforms such as Instagram, Facebook, WhatsApp, TikTok, Twitter, LinkedIn, Pinterest, YouTube, and Snapchat?	3. Yes<br>4. No<br>Refused=-97		GYTS	C
**KAPI918**	During the past 30 days, did you see any advertisements or promotions for tobacco products at points of sale (such as FILL APPROPRIATE COUNTRY EXAMPLES: stores, shops, kiosks, etc.)?	1. I did not visit any points of sale in the past 30 days<br>2. Yes<br>3. No<br>Refused=-97		GYTS	C
**KAPI919**	Would you ever use or wear something that has a tobacco company or tobacco product name or picture on it such as a lighter, t-shirt, hat, or sunglasses?	1. Yes<br>2. Maybe<br>3. No<br>Refused=-97		GYTS	C
**KAPI920**	Has a person working for a tobacco company ever offered you a free tobacco product?	1. Yes<br>2. No<br>Refused=-97		GYTS	C

OPTIONAL MODULES

OPTIONAL MODULE 1: NICOTINE POUCHES

The next section is about "nicotine pouches" such as Zyn, on!, or Velo. These small pouches contain nicotine, and users place them in their mouth. Nicotine pouches are different from other smokeless tobacco products such as snus, dip, or chewing tobacco, because they do not contain any tobacco leaf.

VARNAME	QUESTIONS	CODING CATEGORIES	SKIPS	SOURCE	CORE/OP-TIONAL
**OPM101**	Before today, had you ever seen or heard of nicotine pouches?<br>(USE SHOWCARD)	Yes = 1<br>No = 2<br>Refused = -97	Code 2, -97->OPM103	NYTS	O
**OPM102**	Where did you first see or hear about nicotine pouches?	1. Traditional media: Television, radio, newspapers?<br>2. The internet (online malls, news and non-news web pages)?<br>3. Social media (Facebook, twitter, Instagram, WhatsApp groups)?<br>4. Friends?<br>5. Social gatherings (parties, lounges)<br>96.Others specify_____<br>Refused=-97		NYTS	O
**OPM103**	Have you ever used nicotine pouches, even once or twice?<br>(USE SHOWCARD)?<br><br>FW Note: Ask this question for all the responses in OPM101	Yes = 1<br>No = 2<br>Refused =- 97	Code 2, -97->OPM111	NYTS	O
**OPM104**	How old were you when you first used nicotine pouches?	Age in years___________<br>Refused=-97		NYTS	O

VARNAME	QUESTIONS	CODING CATEGORIES	SKIPS	SOURCE	CORE/OP-TIONAL
**OPM105**	When you first used nicotine pouches, why did you use them?<br><br>(Select one or more)	A. A friend uses them<br>B. A family member uses them<br>C. To try to quit using other tobacco products, such as cigarettes<br>D. They costed less than cigarettes<br>E. They were easier to get than cigarettes<br>F. I had seen people on TV, online, or in movies use them<br>G. They are less harmful than other forms of tobacco, such as cigarettes<br>H. They were available in flavors, such as menthol, mint, candy, fruit, or chocolate<br>I. I could use them unnoticed at home or at school<br>J. I could use them to do tricks<br>K. I was curious about them<br>L. Because I felt anxious, stressed, or depressed<br>M. To get a high or buzz<br>X. Other reason		NYTS	O
**OPM106**	Who were you with when you first used nicotine pouches?<br><br>(Multiple responses allowed)	A. Alone<br>B. With one friend<br>C. With more than one friend<br>D. With a family member<br>E. With more than one family member<br>F. With a new acquaintance<br>X.Others (specify) ____---<br>--------------------			O
**OPM107**	During the past 30 days, on how many days did you use nicotine pouches?	___________days (0-30)<br>Refused=-97	0 -OPM111	NYTS	O

VARNAME	QUESTIONS	CODING CATEGORIES	SKIPS	SOURCE	CORE/OP-TIONAL
**OPM108**	Were any of the nicotine pouches that you used in the past 30 days flavored to taste like menthol, mint, clove or spice, alcoholic drinks, candy, fruit, chocolate, or any other flavor?	Yes = 1<br>No = 2<br>Refused =- 97		NYTS	O
**OPM109**	What flavors were the nicotine pouches that you have used in the past 30 days?<br><br>(Select one or more)	A. Menthol<br>B. Mint<br>C. Clove or spice<br>D. Fruit<br>E. Chocolate<br>F. Alcoholic drinks (such as wine, margarita, or other cocktails)<br>G. Candy, desserts, or other sweets<br>96. Some other flavor not listed here (Specify:______)<br>Refused=-97		NYTS	O
**OPM110**	The last time you used nicotine pouches during the past 30 days, where did you use them?	1. At home<br>2. At a restaurant<br>3. At a bar or club<br>4. School<br>5. At work<br>6. At friends' houses<br>7. In public spaces (e.g. parks, shopping centers, street corners)<br>96. Other. [Please specify]<br>Refused=-97		Nigeria survey, GYTS	O
**OPM111**	During the past 30 days, did anyone refuse to sell you nicotine pouches because of your age?	1. I did not try to buy nicotine pouches during the past 30 days.<br>2. Yes, someone refused to sell me nicotine pouches because of my age.<br>3. No one refused to sell me nicotine pouches because of my age.No, my age did not keep me from buying nicotine pouches.<br>Refused=-97		GYTS	O

VARNAME	QUESTIONS	CODING CATEGORIES	SKIPS	SOURCE	CORE/OP-TIONAL
**OPM112**	During the past 30 days, how did you get your nicotine pouches? (Select one or more)	A. I bought them myself<br>B. I had someone else buy them for me<br>C. I asked someone to give me some<br>D. Someone offered them to me<br>E. I got them from a friend<br>F. I got them from a family member<br>G. I took them from a store or another person<br>X. I got them in some other way (specify: __________)<br>Refused=-97		NYTS	O
**OPM113**	During the past 30 days, where did you buy nicotine pouches?<br><br>(Select one or more)	A. I did not buy nicotine pouches during the past 30 days [EXCLUSIVE RESPONSE]<br>B. I bought them from another person (a friend, family member, or someone else)<br>C. A gas station or convenience store<br>D. A grocery store<br>E. A drugstore<br>F. A mall or shopping center kiosk/stand<br>G. A vending machine<br>H. On the Internet (such as a product website or store website like eBay or Facebook Marketplace)<br>I. Through the mail<br>J. Through a delivery service (such as DoorDash or Postmates)<br>K. A vape shop or tobacco shop<br>X. Some other place not listed here		NYTS	O
**OPM114**	If you bought these products, How much did you spend for a pack?	_________/pack<br>Refused=-97		NYTS	O

VARNAME	QUESTIONS	CODING CATEGORIES	SKIPS	SOURCE	CORE/OP-TIONAL
**OPMN115**	Do you think you will use nicotine pouches in the next 12 months?	1. Definitely yes<br>2. Probably yes<br>3. Probably not<br>4. Definitely not<br>Refused=-97		NYTS	O
**OPMN116**	If one of your best friends were to offer you a nicotine pouch, would you use it?	1. Definitely yes<br>2. Probably yes<br>3. Probably not<br>4. Definitely not<br>Refused=-97		ICT-YATVS	O
**OPMN117**	If one of your close relatives were to offer you a nicotine pouch, would you use it?	1. Definitely yes<br>2. Probably yes<br>3. Probably not<br>4. Definitely not<br>Refused=-97		ICT-YATVS	O

OPTIONAL MODULE 2: CESSATION OF TOBACCO USE

The next questions ask about tobacco use cessation

VARNAME	QUESTIONS	CODING CATEGORIES	SKIPS	SOURCE	CORE/OP-TIONAL
**OPM201**	How easy or difficult would you find it to go without using all tobacco products for as long as a week?	1. Very difficult<br>2. Fairly difficult<br>3. Fairly easy<br>4. Very easy<br>Refused=-97		GYTS	O
**OPM202**	How easy or difficult would you find it to give up using all tobacco products alto-gether if you wanted to?	1. Very difficult<br>2. Fairly difficult<br>3. Fairly easy<br>4. Very easy<br>Refused=-97		GYTS	O
**OPM203**	Are you seriously thinking about quitting the use of all tobacco products? (Please choose the answer that best describes your situation)	1. Yes, during the next 30 days<br>2. Yes, during the next 6 months<br>3. Yes, during the next 12 months<br>4. Yes, but not during the next 12 months<br>5. No, I am not thinking about quitting the use of all tobacco products<br>Refused=-97		NYTS	O
**OPM204**	Thinking about the last time you tried to quit, how long did you stop using all tobacco products?	1. MONTHS_____<br>2. WEEKS______<br>3. DAYS______<br>4. LESS THAN 1 DAY (24HRS)________<br>5. I have never tried to quit<br>Refused= -97	5 -> OPM208	GYTS	O
**OPM205**	Thinking about the last time you tried to quit, did you receive any help or support to stop using tobacco<br><br>(Select all that apply)	A. Yes, from a program or professional<br>B. Yes, from a friend<br>C. Yes, from a family member.<br>D. I tried to quit using medication.<br>E. No, I tried to quit on my own without any help<br>Refused=-97			O

**OPM206**	Thinking about the last time you tried to quit, what was the main reason you decided to stop using all tobacco products?<br><br>(SELECT ONE RESPONSE ONLY)	1. To improve my health<br>2. To save money<br>3. Because my family doesn't like it<br>4. Because my friends do not like it<br>96. Others specify_____<br>Refused=-97		GYTS	O
**OPM207**	Thinking about the last time you tried to quit, when you stopped using all tobacco products, how did you feel about it?	1. It was very difficult<br>2. It was rather difficult<br>3. It was rather easy<br>4. It was very easy<br>Refused=-97		GYTS	O
**OPM208**	Have you ever received help or advice to help you stop using tobacco?<br><br>(SELECT ONLY ONE RE-SPONSE)	A. Yes, from a program or professional<br>B. Yes, from a friend.<br>C. Yes, from a family member.<br>D. Yes, from both programs or professionals and from friends or family members.<br>E. No<br>Refused=-97		GYTS	O

OPTIONAL MODULE 3: SECOND-HAND SMOKE EXPOSURE

The next questions ask about exposure to second-hand tobacco smoke

VARNAME	QUESTIONS	CODING CATEGORIES	SKIPS	SOURCE	CORE/OPTIONAL
**OPM301**	During the past 30 days, on how many days has anyone smoked inside your home, in your presence?	____days<br>Refused=-97		GYTS	O
**OPM302**	How often do you see your father (stepfather or mother's partner) smoking in your home?	1. Don't have/don't see this person<br>2. About every day<br>3. Sometimes<br>4. Never<br>Refused=-97		GYTS	O
**OPM303**	How often do you see your mother (stepmother or father's partner) smoking in your home?	1. Don't have/don't see this person<br>2. About every day<br>3. Sometimes<br>4. Never<br>Refused=-97		GYTS	O
**OPM304**	How often do you see your brother/sister smoking in your home?	1. Don't have/don't see this person<br>2. About every day<br>3. Sometimes<br>4. Never<br>Refused=-97	5 -> OPM208	NYTS	O
**OPM305**	If the response for OPM304 is 2, 3, or 4, is the sibling younger or older?<br><br>Select all that apply	A. Younger<br>B. Older<br>Refused=-97			O
**OPM306**	How often do you see other people smoking in your home?	1. Don't have/don't see these people<br>2. About every day<br>3. Sometimes<br>4. Never<br>Refused=-97		GYTS	O
**OPM307**	During the past 30 days, did you visit any schools?	Yes = 1<br>No = 2<br>Refused =- 97	2 -> OPM309	GATS	O
**OPM308**	Did anyone smoke inside of any school buildings that you visited in the past 30 days?	Yes = 1<br>No = 2<br>Refused =- 97		GATS	O

VARNAME	QUESTIONS	CODING CATEGORIES	SKIPS	SOURCE	CORE/OP-TIONAL
**OPM309**	How often do you see teachers smoking in or around the school?	1. Don't have/not enrolled in school<br>2. About every day<br>3. Sometimes<br>4. Never<br>Refused=-97			O
**OPM310**	During the past 30 days, on how many days has anyone smoked in your presence, inside of any school, university or health facility buildings ?	1. I did not visit a school, university, or health facility during the past 30 days<br>2. I have visited a school, university, or health facility but no one smoked in my presence<br>3. 1 to 2 days<br>4. 3 to 4 days<br>5. 5 to 6 days<br>6. 7 days<br>7. More than 7 days<br>Refused=-97		GYTS	O
**OPM311**	During the past 30 days, on how many days has anyone smoked in your presence, inside any public transportation vehicles, such as trains, buses, or taxicabs?	1. I did not use public transportation during the past 30 days<br>2. I used public transportation but no one smoked in my presence<br>3. 1 to 2 days<br>4. 3 to 4 days<br>5. 5 to 6 days<br>6. 7 days<br>7. More than 7 days<br>Refused=-97		GYTS	O
